# Physics-informed neural network simulation of thermal cavity flow

**DOI:** 10.1038/s41598-024-65664-3

**Published:** 2024-07-02

**Authors:** Eric Fowler, Christopher J. McDevitt, Subrata Roy

**Affiliations:** Applied Physics Research Group, University of Florida, Gainesville, Florida 32611 United States

**Keywords:** Mechanical engineering, Hydrogen storage, Batteries, Fluid dynamics, Biological physics

## Abstract

Physics-informed neural networks (PINNs) are an emerging technology that can be used both in place of and in conjunction with conventional simulation methods. In this paper, we used PINNs to perform a forward simulation without leveraging known data. Our simulation was of a 2D natural convection-driven cavity using the vorticity-stream function formulation of the Navier-Stokes equations. We used both 2D simulations across the *x* and *z* domains at constant Rayleigh (*Ra*) numbers and 3D simulations across the *x*, *z* and *Ra* domains. The 3D simulation was tested for a PINN’s ability to learn solutions in a higher-dimensional space than standard simulations. The results were validated against published solutions at *Ra* values of 10$^{3}$, 10$^{4}$, 10$^{5}$, and 10$^{6}$. Both the 2D simulations and 3D simulations successfully matched the expected results. For the 2D cases, more training iterations were needed for the model to converge at higher *Ra* values (10$^5$ and 10$^6$) than at lower *Ra* (10$^3$ and 10$^4$) indicating increased nonlinear fluid-thermal coupling. The 3D case was also able to converge but, but it required more training than any of the 2D cases due to the curse of dimensionality. These results showed the validity of standard simulations via PINNs and the feasibility of higher-order parameter space solutions that are not possible using conventional methods. They also showcased the additional computational demand associated with increasing the dimensionality of the learned parameter space.

## Introduction

Due to the computational capabilities of the modern hardware, the computational fluid dynamics (CFD) field has seen rapid progress^1^ and has provided immense value for engineers facing modern challenges. In recent years, the popularity of machine learning and the production of powerful and accessible computing tools, such as GPUs and TPUs, have provided new opportunities to expand upon existing CFD methods. One such method is approximating the solutions of partial differential equations (PDEs) using artificial neural networks, which are commonly referred to as “black boxes”^2^ due to their ability to approximate *correct* solutions using learned weights that may not make logical sense to a human observer. An application of neural networks to solve PDEs is more specifically referred to as physics-informed neural networks (PINNs)^3^. PINNs can be used to find solutions to equations that reflect real-world physics, such as the Navier-Stokes equations, but they can also be applied to any dynamical system^4^ modeled using partial differential equations, such as the Black-Scholes model^5^ in finance.

PINNs are flexible simulation tools that can be used to model various physical phenomena with limited changes to their code. Forward simulation, the method explored in this paper, finds the solution of a system using known initial and boundary conditions as well as the known governing equations. Neural networks are powerful tools for learning from data, which means that existing data can be added to a PINN to have it learn to match the existing data as an additional boundary condition. Adding this extra data as a learned boundary condition requires minimal changing of the network, which consists of defining the boundary condition as a loss term and including it in the backpropagation step. Another use of PINNs is an inverse simulation using known data^4,6^. For inverse learning, PINNs will learn to match the data and use the known parts of the governing equations to solve for the unknown parameters. For example, if the velocities of a flow are known and the density and viscosity are unknown, then the PINN can learn to fit these velocities and use the Navier-Stokes equations to determine the unknown density and viscosity parameters^7^. As long as an adequate loss function can define a PINN’s performance and the PINN has enough nodes and layers, it can be used to learn any set of desired outputs from its given inputs. In this paper, PINNs are employed and tested for performance on a heat transfer benchmark, specifically the buoyancy-driven thermal cavity.

Albeit exhaustively studied, the rectangular thermal cavity remains an excellent example of a purely buoyancy-driven flow that has practical applications in thermal insulation, heating and ventilation of buildings, smoke stacks from fire, solar collectors and the cooling of electronic equipment^8–10^. Specifically, the 2D natural convection in a square cavity has served as a CFD benchmark for many years and has validated a wide spectrum of techniques, such as finite-difference^11^, differential quadrature^12^, lattice Boltzmann^13^, and Lagrange interpolating polynomial^14^. It has also been extensively studied experimentally^14,15^. Given the availability of these well-documented solutions, the thermal cavity problem is an excellent benchmark for testing the performance of our PINN.

One downside of many standard numerical simulations is that they can require careful attention to meshing^16^. In comparison, PINNs can be trained on random collocation points in the desired domain, thus greatly facilitating the treatment of complex geometries. Also, training can often be done using batch learning, where the neural network’s performance at each point is evaluated simultaneously and the average loss across all points is used to train the network. This means that PINNs are not dependent on any sort of meshing, and the main priority when generating training points is sufficiently representing the parameter space of the problem to ensure convergence towards the global minimum^17^. Certain random sampling methods, such as Latin hypercube sampling (LHS) can be used for random point generation, but they may not be necessary for solution convergence. This paper used uniform random sampling for point generation. An additional advantage of PINNs is that they have been shown to be proficient at learning parametric solutions to PDEs^18,19^.Here, while the online training time of the PINN can be substantial, once trained a single PINN can be used to predict solutions of the PDEs across a broad range of parameters. Combined with the fast online execution time of a neural network this property can be used to develop rapid surrogate models of PDEs^20^. Such surrogate models facilitate the rapid exploration of the solution space of a given system of PDEs^21,22^, as well as enable the development of integrated descriptions of multiphysics systems^23^.

Traditional CFD codes can easily simulate the 2D thermal cavity, but the limitation is that each simulation is only at a set *Ra* number. Any sort of information about the transition from one *Ra* value to another is unknown, and the only way to evaluate the range of solutions between two *Ra* values is to individually simulate the solution at each desired *Ra* in that range. In contrast, using a 3D PINN with inputs of (*x*, *z*, *Ra*) will enable near-instantaneous prediction of solutions at any desired *Ra* value within the lower and upper *Ra* limits the PINN was trained on. This can help give much more insight into specific flow features, such as the movement of the location of maximum temperature^11–14^, without needing to continuously run simulations to evaluate a new *Ra* value. This increased 3D parameter space simulation may take a significant amount of time to train, such as on the order of hours or days, but evaluating the model after training can be done in real-time, such as on the order of milliseconds^24^ or possibly even nanoseconds^25,26^.

The primary objective of this research is to validate PINNs on simulations with known solutions^11^. Existing data was used to validate the results, but it was not used to train the models. Once the code is validated, it could be applied to more challenging scientific and engineering problems, such as higher *Ra* simulations, more complicated boundary conditions, and flows with discontinuities.

The second objective is to identify the advantages and disadvantages of PINNs as an alternative CFD method. Runtime is typically an important metric for evaluating numerical code, but comparing the total development time for creating and running a successful PINN or CFD code might be of more value to researchers. However, comparing the meshless random sampling of a PINN to the effort needed to construct a sufficient CFD grid is not easily quantified. Therefore, the more unique capabilities of PINNs, such as learning across all *Ra* values simultaneously and evaluating gradients using graphs, will be discussed more heavily.

## Problem description

These simulations used a two-dimensional Boussinesq approximation of a fluid in a vertical square cavity with unit lengths of 1 in the horizontal (*x*) and vertical (*z*) directions (and assumed infinite depth in the *y* direction). Note that the use of the term “3D” refers to simulation over (*x*, *z*, *Ra*) and not the physical dimensions of (*x*, *y*, *z*). For the Boussinesq approximation, the two parameters that can define the flow are the Prandtl number (*Pr*) and the Rayleigh number (*Ra*).

The *Pr* number is a dimensionless value of a fluid that relates its viscous diffusivity to its thermal diffusivity. This relationship is typically given using Eq. (1)

1 $$\begin{aligned} Pr = \frac{\nu }{\alpha } = \frac{{c_p}\mu }{k} \end{aligned}$$

where $\nu$ is the kinematic viscosity, $\alpha$ is the thermal diffusivity, $c_p$ is the specific heat, $\mu$ is the dynamic viscosity, and *k* is the thermal conductivity. The conventional *Pr* number for the thermal cavity simulation is 0.71^11–14^, which is the *Pr* value for air at standard temperature and pressure, so that value was also chosen for all our simulations.

The *Ra* number is a dimensionless value that relates the buoyancy of a fluid to its thermal diffusivity. It can be calculated as the product of the Grashof number (*Gr*) with *Pr*. This relationship is defined using Eq. (2)

2 $$\begin{aligned} Ra = GrPr = \frac{{\rho }{\beta }{\Delta }Tl{^3}g}{{\nu }{\alpha }} \end{aligned}$$

where $\rho$ is density, $\beta$ is the coefficient of thermal expansion, ${\Delta }T$ is the temperature difference across a distance *l*, and *g* is the acceleration due to gravity. As *Pr* will remain constant at 0.71 for the simulations, *Ra* will be the parameter that varies in the simulations. By extension, this means that the *Gr* number will be what is manipulated to vary *Ra*, but all discussion will only include *Ra* since the corresponding *Gr* number can be easily calculated.

The PINN outputs were the stream function (${\psi }$) and reference temperature (*T*). All other values were derived derivatives of these outputs with respect to the inputs. The u-velocity described flow in the horizontal direction, and the w-velocity described the flow in the vertical direction.

**Figure 1:**
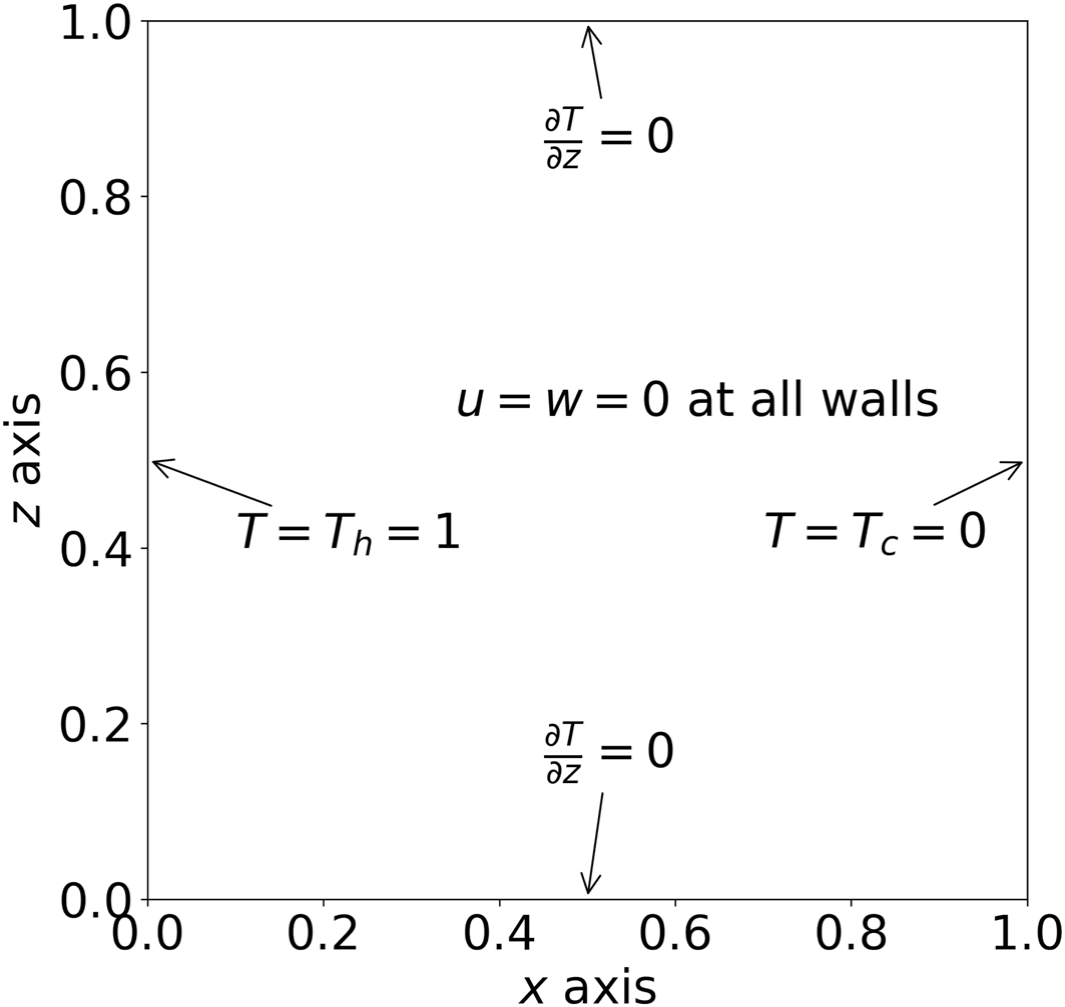
Problem Geometry and Boundary Conditions.

### Boundary conditions

The no-slip condition was enforced for both the u and w velocities at all four walls. The left wall was defined as a constant temperature $T_h$ and was the maximum temperature of the simulation. The right wall was set at a constant temperature $T_c$ and was the coldest temperature of the simulation. The reference temperature *T* was calculated using $T = {\frac{T'-T_c}{T_h-T_c}}$ where $T'$ was the absolute temperature. By using $T_h = 1$ and $T_c = 0$, this condition was easily satisfied to enable simulation over just the reference temperature *T* rather than needing to convert to absolute values. The top and bottom walls were insulated, which meant $\frac{\partial {T}}{\partial {z}}=0$ along those locations. These boundary conditions are shown in Fig. 1.

The boundary conditions were enforced using a combination of hard constraint transformations and learned constraints. The no-slip velocity conditions and the constant wall temperatures were enforced using hard constraints, but the insulated boundary conditions were enforced as learned constraints (i.e. by adding a penalty term to the loss function).

### Governing equations

Our solution followed the non-dimensional stream function-vorticity (${\psi }$-${\zeta }$) definition for a Boussinesq fluid. These equations are shown in Eqs. (3)–(5).

3 $$\begin{aligned} {{\partial }\over {{\partial }x}}(u\zeta )+{{\partial }\over {{\partial }z}}(w\zeta ) =Pr{\nabla }^{2}{\zeta }+RaPr{{\partial }T\over {{\partial }x}} \end{aligned}$$

4 $$\begin{aligned} 0 = {\nabla }^{2}{\psi }+{\zeta } \end{aligned}$$

5 $$\begin{aligned} {{\partial }\over {{\partial }x}}(uT)+{{\partial }\over {{\partial }z}}(wT)={\nabla }^{2}T \end{aligned}$$

The value of $Pr=0.71$ was kept constant across all simulations.

By using a smooth activation function, the PINN was continuous and differentiable at each point. This meant that we could use the properties of graph networks to exactly evaluate the derivatives at each point without the need for a finite-difference scheme. Since graph networks make derivatives straightforward to calculate, the graphs for all the derivatives calculated in the governing equations were shown in the results as extra data. This includes fourth-order derivatives of the stream function, which serve as evidence of a PINN’s ability to effectively learn high-order derivatives.

### Hard boundary constraints

To enforce the no-slip condition, $\psi$ needed to have a derivative of 0 with respect to each of *x* and *z* at all the walls. This was accomplished using two equations

6 $$\begin{aligned} bcv = 16(x)(1-x)(z)(1-z) \end{aligned}$$

and

7 $$\begin{aligned} \psi = (bcv)^2{\psi }_0 \end{aligned}$$

where ${\psi }_0$ was the original output of the PINN and $\psi$ was the final output of the model.

The constant temperature conditions $T_h$ and $T_c$ were enforced on the reference temperature *T* by the equation

8 $$\begin{aligned} T = 1-x+sin({\pi }x)T_0 \end{aligned}$$

where $T_0$ was the output of the PINN. This enforced the reference temperature *T* to be 1 when $x=0$ and 0 when $x=1$, which meant it matched with the desired $T_h$ and $T_c$ for the model.

### Loss equations

The loss equations were defined using the mean-squared error (MSE). MSE was chosen because it is differentiable, which is necessary for backpropagation. The cumulative loss function used is given by Eq. (9)

9 $$\begin{aligned} Loss = Loss_{PDE}+Loss_{BC} \end{aligned}$$

which was defined using Eqs. (11) and (10)

10 $$\begin{aligned} Loss_{PDE} = {1\over {N_{PDE}}}\sum _{i=1}^{N_{PDE}}\left[ {\left( {\frac{{f_{1} (x_{i} ,y_{i} ,Ra_{i} )}}{{Ra_{i} }}} \right)^{2} } +{(f_{2}(x_{i},y_{i},Ra_{i}))}^2+{(f_{3}(x_{i},y_{i},Ra_{i}))}^2\right] \end{aligned}$$

where $f_{1}$ is given by Eq. (12), $f_{2}$ is given by Eq. (13), and $f_{3}$ is given by Eq. (14).

11 $$\begin{aligned} Loss_{BC} = {1\over {N_{BC}}}\sum _{i=1}^{N_{BC}}(t_{i}-y_{i})^2 \end{aligned}$$

12 $$\begin{aligned} f_{1}(x,y,Ra) = {{\partial }\over {{\partial }x}}(u\zeta )+{{\partial }\over {{\partial }z}}(w\zeta )-Pr{\nabla }^{2}{\zeta } -RaPr{{\partial }T\over {{\partial }x}} \end{aligned}$$

13 $$\begin{aligned} f_{2}(x,y,Ra) = {\nabla }^{2}{\psi }+{\zeta } = 0 \end{aligned}$$

14 $$\begin{aligned} f_{3}(x,y,Ra) = {{\partial }\over {{\partial }x}}(uT)+{{\partial }\over {{\partial }z}}(wT)-{\nabla }^{2}T \end{aligned}$$

where $t_i$ is the target value at the boundary and $y_i$ is the predicted value at the boundary. The only boundary condition enforced via this method is the insulated boundary at the top and bottom walls, where $\frac{\partial {T}}{\partial {z}} = 0$, thus making $t_{i} =0$ for all *t*. The residuals of Eqs. (3)–(5) are represented as $f_1$, $f_2$, and $f_3$, respectively. Due to all the derivatives being calculated using graphs (which enables us to compute derivatives algebraically rather than approximate them numerically), the loss related to Eq. (4) would be evaluated as identically zero, so it was removed to avoid unnecessary computation. In Eq. (10) the loss for Eq. (3) was divided by the *Ra* number before the residual was squared to prevent the equation from dominating the loss terms due to the large *Ra* value.

### PINN training specifics

As with most machine learning methods, neural networks perform better when each input *X* is transformed by some function $f:X \mapsto [0,1]$ for all *X*^27^. The *x* and *z* values did not require transformation, as they already satisfied this condition. However, for the (*x*, *z*, *Ra*) case, the input values of *Ra* needed to be transformed from $Ra_{true} \in [ 10^3,10^6]$ to the desired range of $Ra_{input}\in [0,1]$. The chosen function to make this transformation was

15 $$\begin{aligned} Ra_{input}=f(Ra_{true}) = {\log _{10}(Ra_{true})-3\over {3}}. \end{aligned}$$

For the 2D cases, the collocation points for training Eq. (10) were randomly selected from a uniform distribution. 10,000 were randomly selected in the domain, and 1000 were randomly selected for each wall. The separate collocation points for training the insulation boundary conditions in Eq. (11) were randomly selected from a uniform distribution along the top and bottom walls. Each wall had an additional 1000 points randomly assigned to it. All simulations were performed using the same random seed to ensure the experiment is easily repeatable. It also makes runtime and performance easier to compare between runs.

For the 3D case, the collocation points for training Eq. (10) were randomly selected from a uniform distribution. 50,000 were randomly selected in the domain, and 3000 were randomly selected for each wall. The collocation points for training the insulation boundary conditions in Eq. (11) were randomly selected from a uniform distribution along the top and bottom walls. Each wall had an additional 3000 points randomly assigned to it. All simulations were assigned random points from a uniform distribution between values 0 and 1 for the transformed value of *Ra*. While the choice of a uniform random distribution of points is by far the most common choice used when training PINNs, recent work suggests potential improvements are possible by considering distinct static training point distributions, or using adaptive sampling techniques^28^. Future work will investigate whether these alternate point distributions are able to improve the training in the context of the thermal driven cavity.

One advantage of training using neural networks was the availability of graphs^29^. Graph networks enable the exact calculation of the derivatives of a function, referred to as “automatic differentiation”^30^ which meant that all the derivatives for the Navier-Stokes equations could be calculated exactly and without the need for finite-difference methods. All simulations were performed using TensorFlow, so TensorFlow GradientTape was employed for all derivative calculations.

One of the most important aspects of PINN training was picking the correct optimizer. Many conventional neural nets perform well using the ADAM^31^ and stochastic gradient descent (SGD) optimizers, however, these optimizers have been overshadowed by the BFGS optimizer and its limited-memory version, the L-BFGS optimizer, for PINN simulations^32^. The BFGS optimizer is a Quasi-Newton Method^33^ that approximates the inverse of the Hessian matrix^33,34^ of the loss function with respect to the weights of the PINN. This optimizer, which has a computational complexity of $O(n^{2})$, where *n* is the number of weights, places a demanding memory constraint on the PINN that can easily consume all 80 GB of VRAM of an A100 GPU. Despite the resource demands of the BFGS optimizer, it was chosen over the ADAM optimizer for its better ability to minimize the loss function.

The final results were evaluated at selected points for the values of $|\psi |$, *u*, and *w* to compare to known results^11^.

### PINN hyperparameters

The 2D (*x*, *z*) simulations corresponded to a network architecture of 2 input neurons for the first layer, 7 hidden layers of 64 neurons with *tanh* activation functions, and an output layer of 2 neurons with no activation functions.

The 3D (*x*, *z*, *Ra*) simulations were trained using a network with an input layer of 3 neurons, 7 hidden layers of 50 neurons with *tanh* activation functions, and an output layer of 2 neurons with no activation functions. The increase from 2 dimensions to 3 meant that more training points were necessary to properly sample the desired (*x*, *z*, *Ra*) domain. An increased number of inputs also meant that the number of weights in the PINN increased, which would heavily affect the memory usage of the BFGS optimizer. This would have required more memory than was available on our A100 GPU, so the number of neurons had to be decreased while adding more collocation points for proper sampling.

The number of training iterations changed for each case due to large variations in model performance at each *Ra* value. Also, due to the line search algorithm in the optimizer, the number of requested training iterations and the number of reported iterations (the total number of loss function evaluations) recorded in plots often differ by factors of $\approx 2$. To avoid any potential confusion, the runtimes for training each model are also given as a primary metric for comparing performance. Note that the entire dataset was evaluated in one batch, so one iteration was the same as one epoch.

### Hardware

The simulations were performed on the University of Florida HiPerGator supercomputer using Nvidia Ampere A100 GPUs with 80 GB of memory. Due to the BFGS optimizer used from TensorFlow Probability not supporting distributed training, only 1 GPU was used to train each model.

### Data availability

The Python codes with models that generated datasets presented in this paper are available in the GitHub repository [https://github.com/cmcdevitt2/ThermalCavity].

## Results

The results for each case are shown along with the loss history of their training and a description of their performance in comparison to known values^11^ are shown in Table 1 and Table 2. For comparison with Ref.^11^, the value of *u* was the maximum value in the vertical slice at $x=0.5$, and the value of *w* was the maximum value in the horizontal slice at $z=0.5$. Our results evaluated *u* and *w* at the same locations as given in the literature^11^. The location for the evaluation of $|\psi |$ was defined as (0.5, 0.5).

### 2D simulations

The results for the 2D simulations are tabulated in Tables 1 and 2, the contour plots are shown in Figs. 2, 3, 4 and 5 and plots of the losses are shown in Figs. 6, 7, 8 and 9.

The models for all 4 cases produced predictions that matched the reference solutions^11^. The soft-enforced boundary condition of $\frac{{\partial }T}{{\partial }z}$ at the top and bottom walls held true for all cases, which could be seen in both the contour plots and in the “Loss_BC” term in the loss plots.

All loss plots show a similar pattern of high variance in the loss before eventually converging more smoothly. When comparing these loss plots, as the value of *Ra* increases, the length of this high-variance region increases. The $Ra=10^{3}$ and $Ra=10^{4}$ cases both converged at similar rates, so they received the same amount of training and finished with their losses at the same order of magnitude. In contrast, the $Ra=10^5$ and $Ra=10^6$ required more training in comparison to the $Ra=10^{3}$ and $Ra=10^{4}$ cases. Both were trained for about 4 hours, and the $Ra=10^5$ case finished with a lower MSE loss than the $Ra=10^6$ case. Upon inspection of the higher *Ra* models during training, it seemed that the $Ra=10^5$ and $Ra=10^6$ models were converging towards incorrect solutions before eventually converging towards the correct solutions. Although these black-box models were eventually able to converge, this optimization challenge significantly increased the training time of these larger *Ra* cases in comparison to the lower *Ra* cases.

**Table 1:** Data comparison of PINN predicted values for cavity with Ref.^11^ at $Ra=10^{3}$, $10^{4}$, $10^{5}$, and $10^{6}$. The order of magnitude of the MSE loss and the runtime are also tabulated.

Parameter	$Ra=10^3$	$Ra=10^4$	$Ra=10^5$	$Ra=10^6$
*Nu*	a	b	c	d
$|\psi |_{ref}$ location (*x*, *z*)	(0.5, 0.5)	(0.5, 0.5)	(0.5, 0.5)	(0.5, 0.5)
$|\psi |_{ref}$	1.174	5.071	9.111	16.32
$|\psi |_{pred}$	1.173	5.065	9.116	16.38
$u_{ref}$ location (*x*, *z*)	(0.5, 0.813)	(0.5, 0.823)	(0.5, 0.855)	(0.5, 0.850)
$u_{ref}$	3.649	16.178	34.73	64.63
$u_{pred}$	3.643	16.135	34.74	64.81
$w_{ref}$ location (*x*, *z*)	(0.178, 0.5)	(0.119, 0.5)	(0.066, 0.5)	(0.0379, 0.5)
$w_{ref}$	3.697	19.617	68.59	219.36
$w_{pred}$	3.691	19.590	68.64	220.52
Loss order of magnitude	$10^{-4}$	$10^{-4}$	$10^{-7}$	$10^{-6}$
Runtime	24 min	24 min	4 h 2 min	3 h 55 min

**Table 2:** Nusselt number (*Nu*) values calculated from 2D PINNs compared with reference^11^ at $Ra=10^{3}$, $10^{4}$, $10^{5}$, and $10^{6}$.

	$Ra=10^3$	$Ra=10^4$	$Ra=10^5$	$Ra=10^6$
PINN				
$Nu_{0}$	1.118	2.245	4.522	8.825
$Nu_{min}$	0.691	0.585	0.728	0.979
$y_{Nu,min}$	0.996	1	1	1
$Nu_{max}$	1.506	3.531	7.720	17.545
$y_{Nu,max}$	0.088	0.144	0.082	0.039
$Nu_{\frac{1}{2}}$	1.118	2.245	4.522	8.825
$\bar{Nu}$	1.118	2.244	4.519	8.814
Ref.^11^				
$Nu_{0}$	1.117	2.238	4.509	8.817
$Nu_{min}$	0.692	0.586	0.729	0.989
$z_{Nu,min}$	1	1	1	1
$Nu_{max}$	1.505	3.528	7.717	17.925
$z_{Nu,max}$	0.092	0.143	0.081	0.0378
$Nu_{\frac{1}{2}}$	1.118	2.243	4.519	8.799
$\bar{Nu}$	1.118	2.243	4.519	8.800

**Figure 2:**
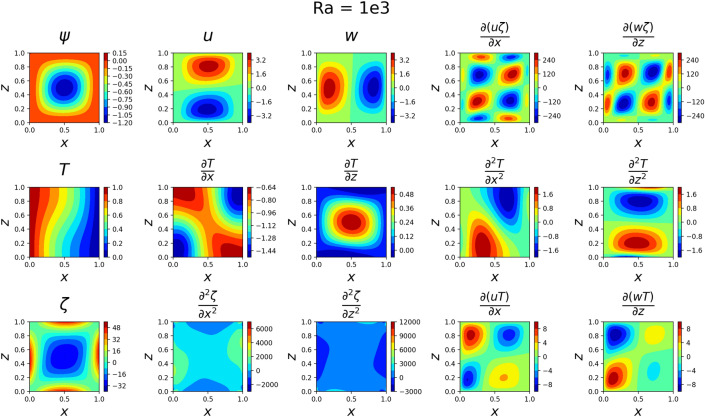
Outputs of 2D $Ra=10^3$ simulation. The stream function looks somewhat circular around (0.5, 0.5), the *u* and *w* velocity magnitudes are low, and the temperature gradient varies little across *z*.

**Figure 3:**
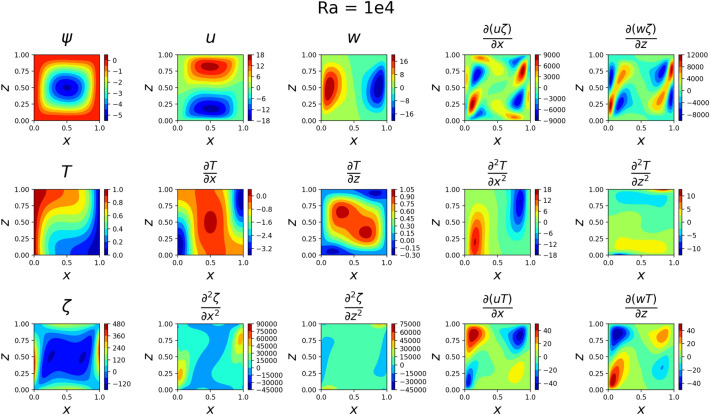
Outputs of 2D $Ra=10^4$ simulation. Some of the more basic shapes from the $Ra=10^{3}$ case have started to shift. *T* changes more over *z* and $\frac{\partial {T}}{\partial {z}}$ has split into two maximum regions.

**Figure 4:**
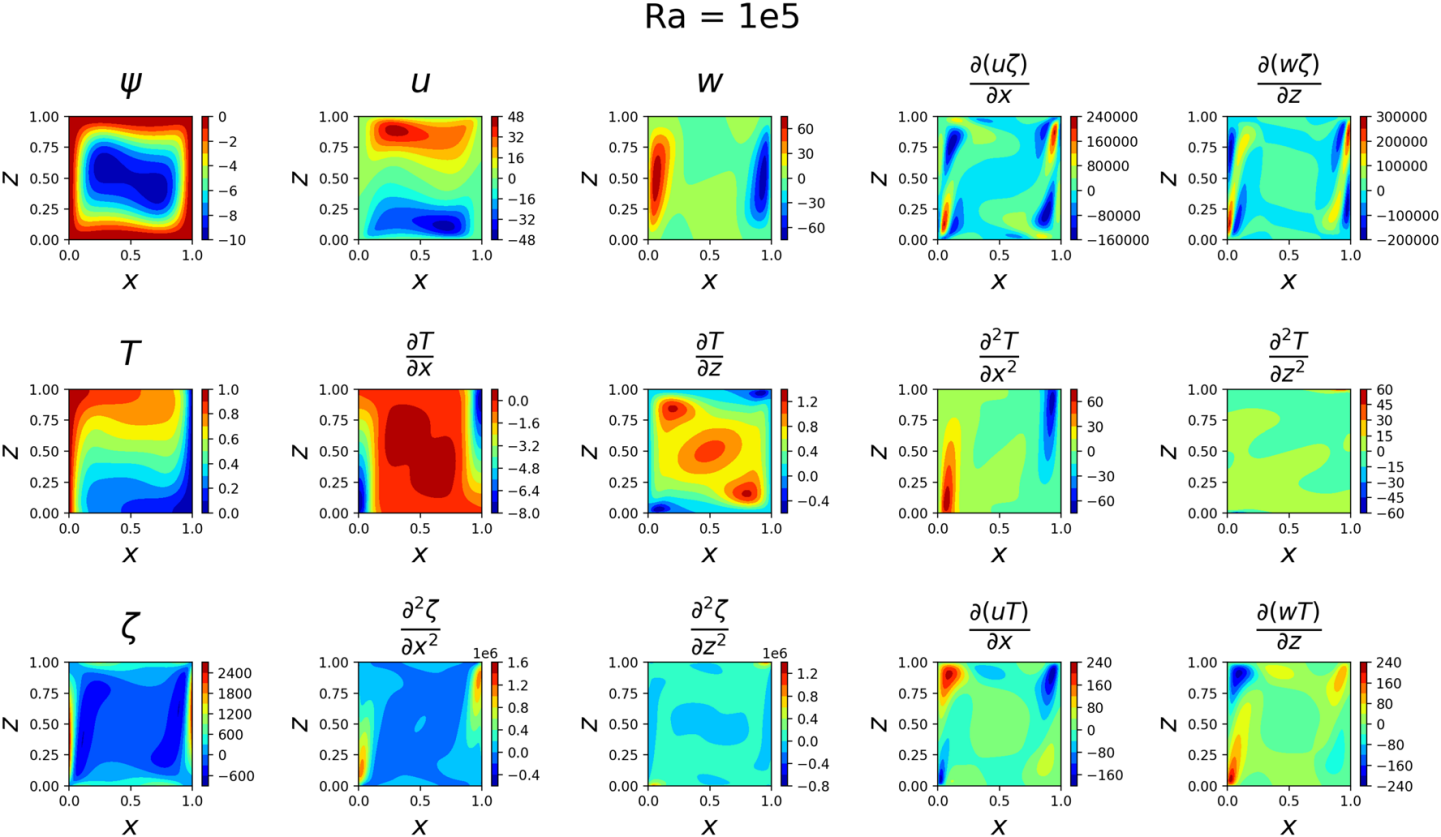
Outputs of 2D $Ra=10^5$ simulation. *T* now heavily changes across *z*, and *w* seems to have developed jet-like flows near the side walls. The flow velocities are much larger now than before.

**Figure 5:**
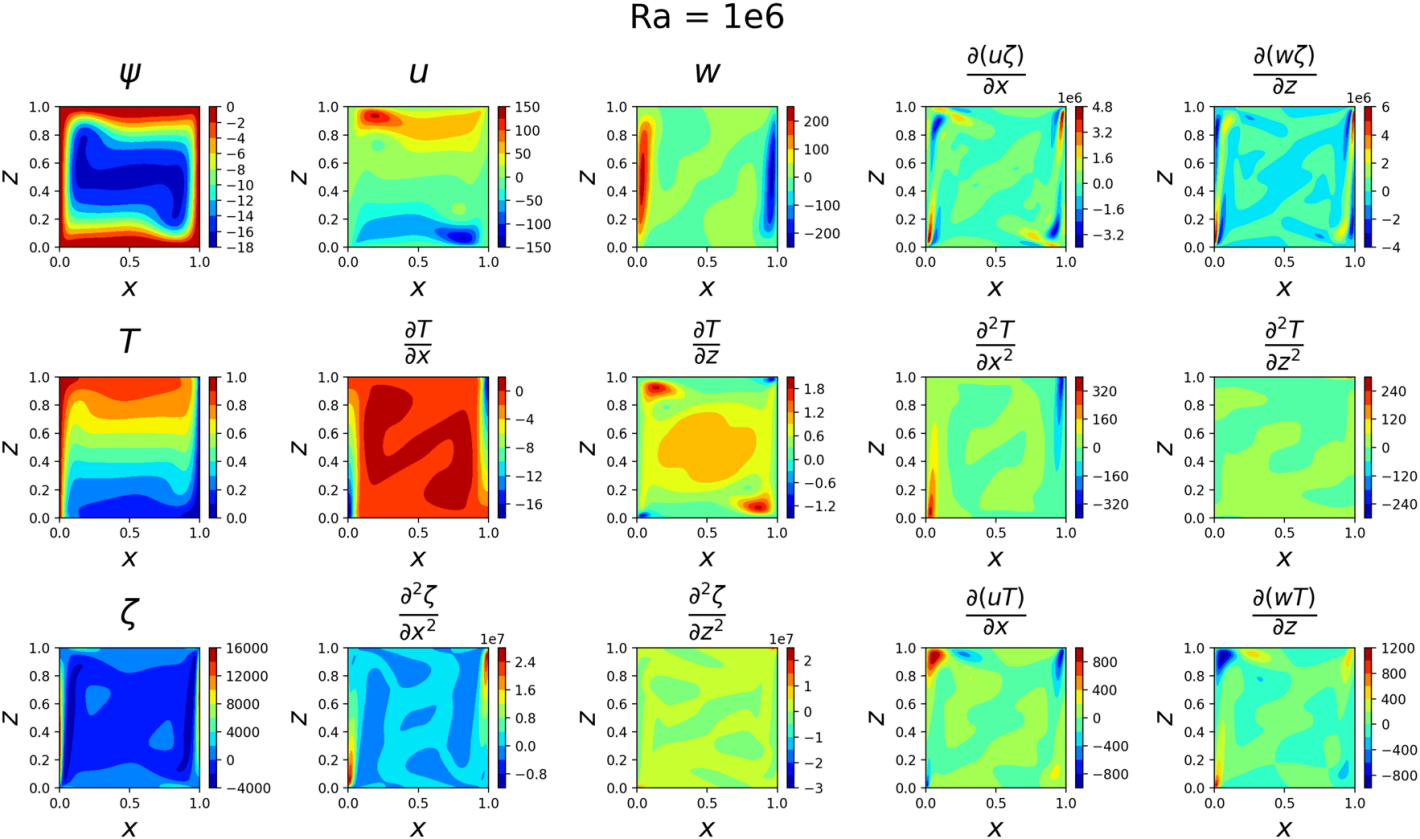
Outputs of 2D $Ra=10^6$ simulation. The flow at the side walls has become even more jet-like in *w*. The velocities have increased in magnitude by about 4 times what they were in the $Ra=10^{5}$ case. Some of the plots on the bottom row have such large variances in magnitude that features are largely indistinguishable.

**Figure 6:**
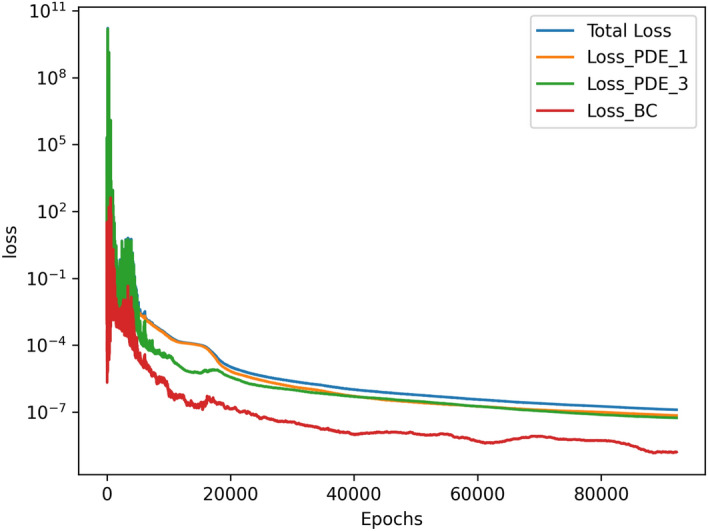
Loss graph of $Ra=10^3$ simulation.

**Figure 7:**
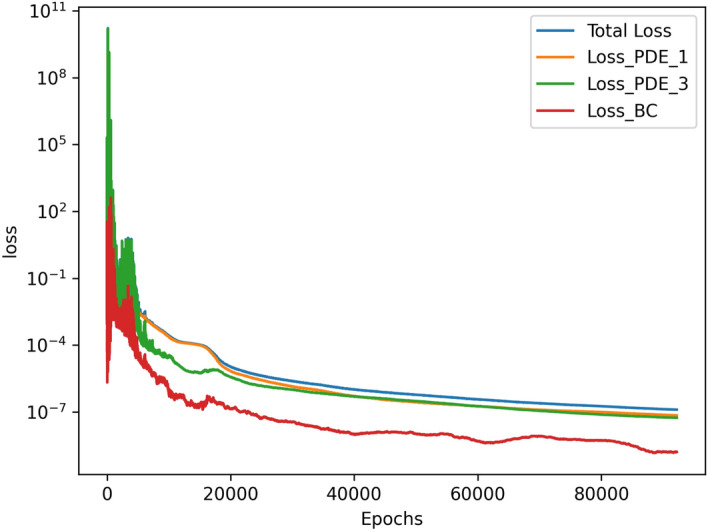
Loss graph of $Ra=10^4$ simulation.

**Figure 8:**
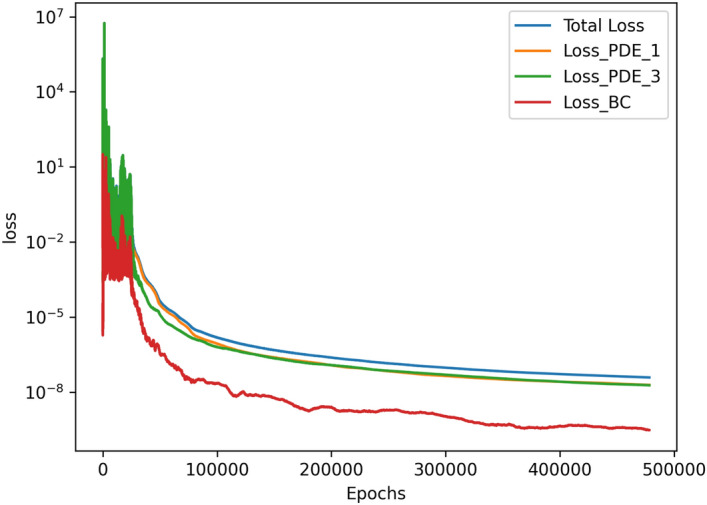
Loss graph of $Ra=10^5$ simulation.

**Figure 9:**
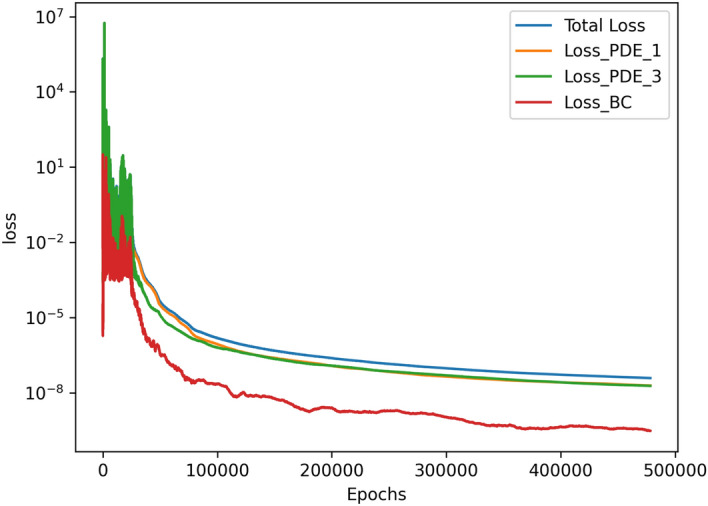
Loss graph of $Ra=10^6$ simulation.

**Figure 10:**
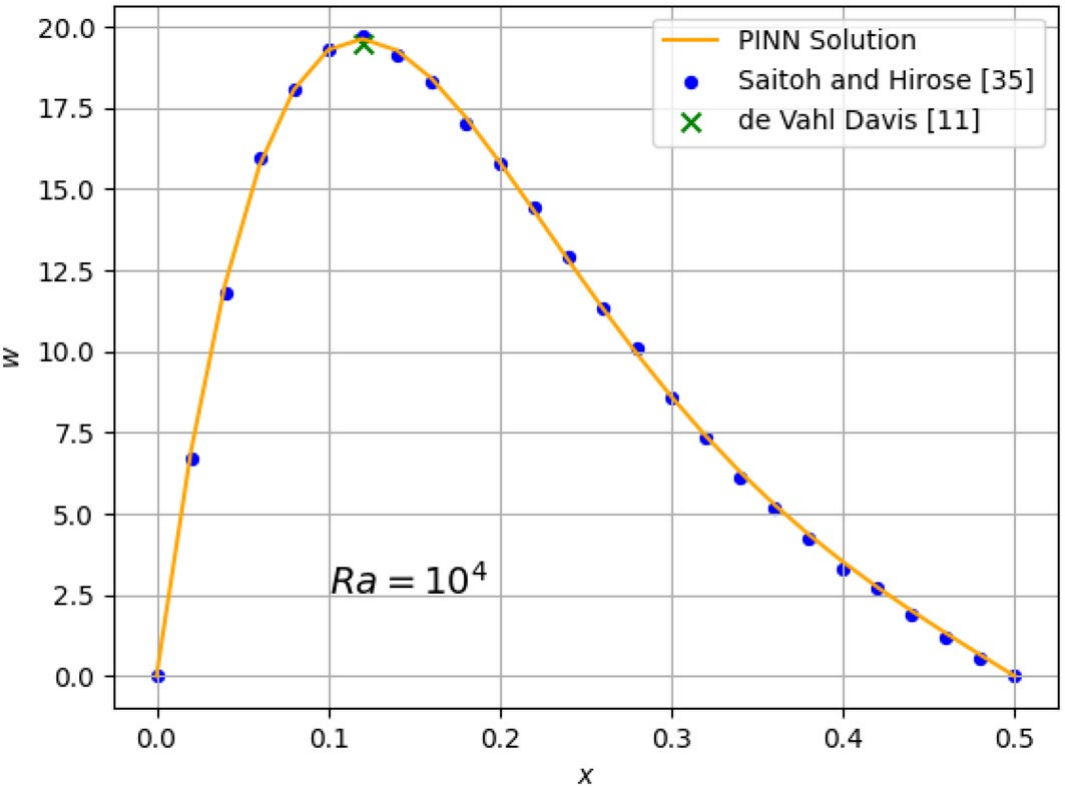
Comparison of 2D $Ra=10^{4}$ prediction of *w* at $z=0.5$ with published data points^11,35^.

**Figure 11:**
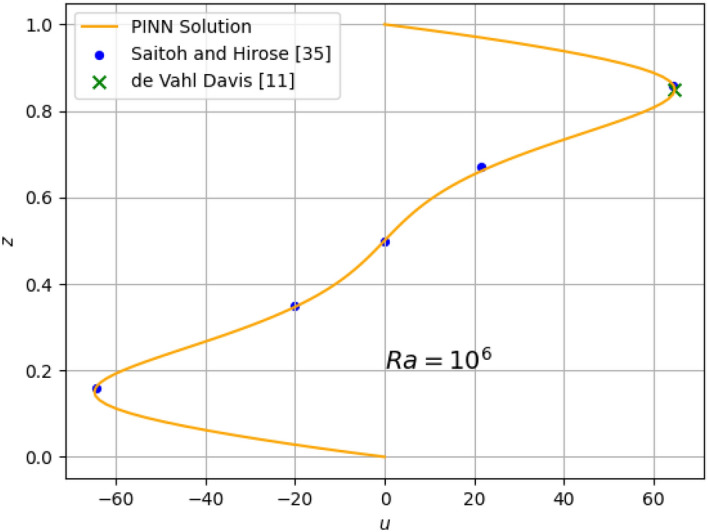
Comparison of 2D $Ra=10^{6}$ prediction of *u* at $x=0.5$ with published data points^11,35^.

**Figure 12:**
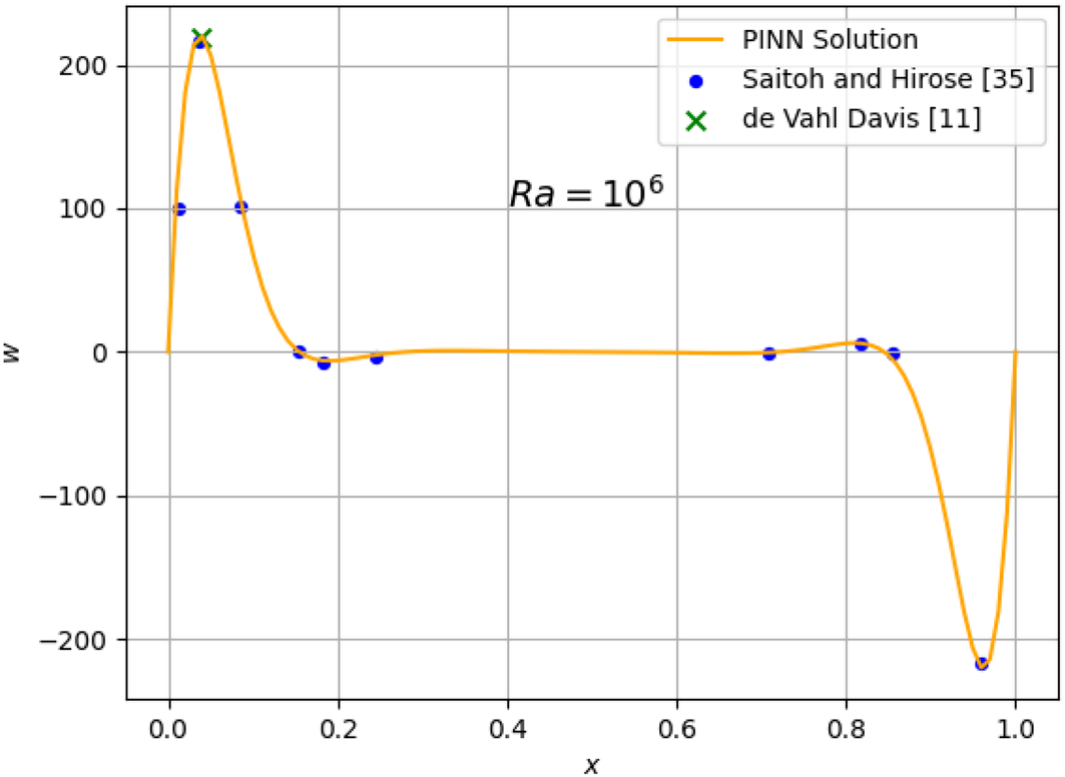
Comparison of 2D $Ra=10^{6}$ prediction of *w* at $z=0.5$ with published data points^11,35^.

The 2D results for *w* at the $z=0.5$ were compared against published data^11,35^ in Fig. 10 for a quantitative validation of the PINN’s accuracy at $Ra=10^{4}$. Additionally, midplane plots of *u* and *w* Figs. 11 and 12 at $Ra=10^{6}$ were plotted against reported data^11,35^.

### 3D simulation

**Figure 13:**
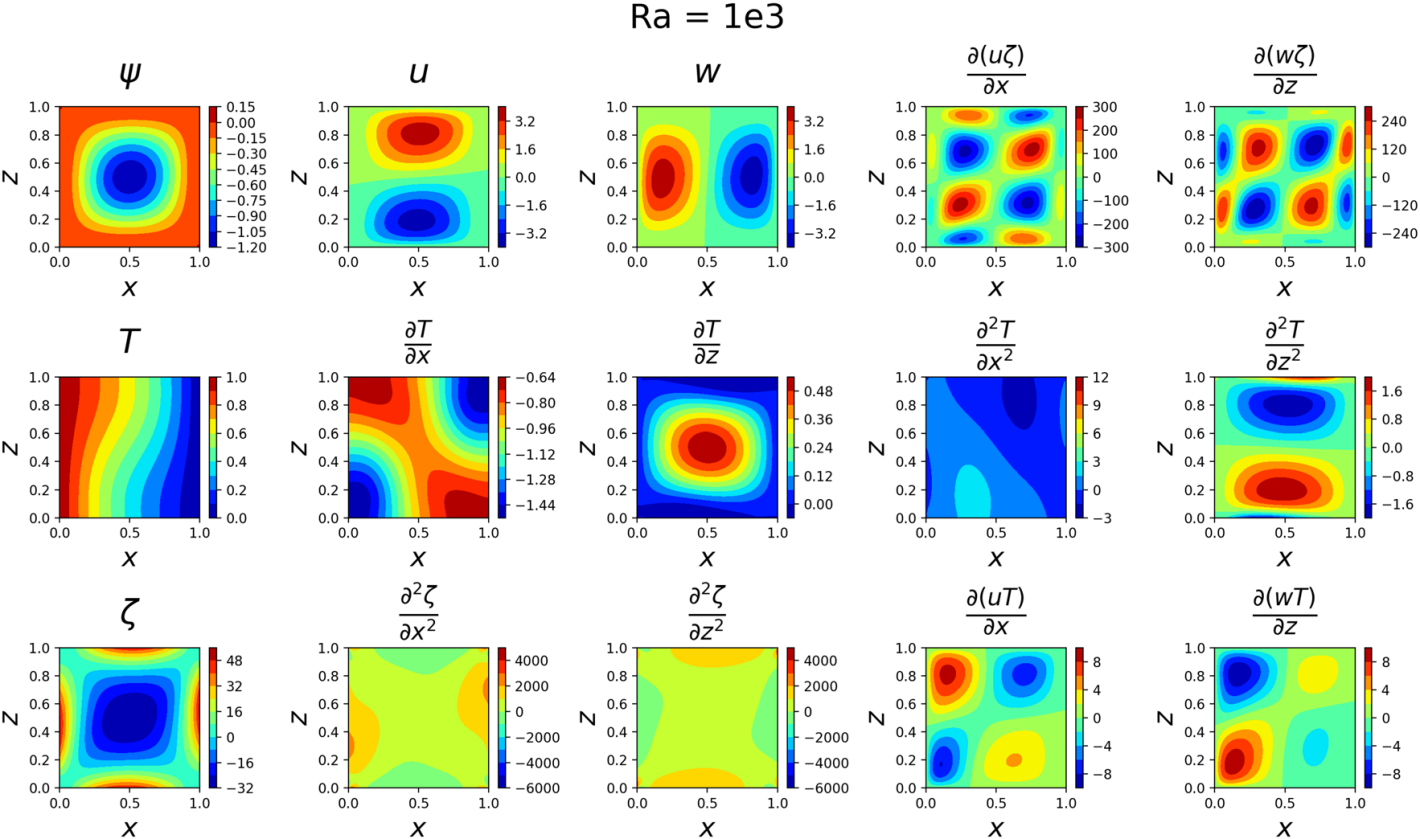
Outputs of 3D $Ra=10^3$ simulation. Most plots match the 2D case well except for some of the second-order derivatives.

**Figure 14:**
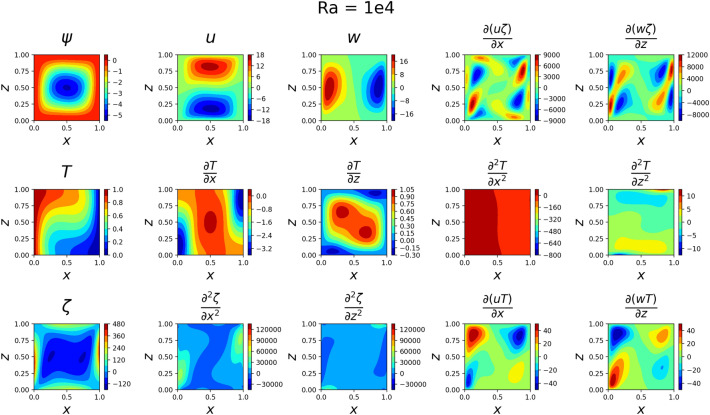
Outputs of 3D $Ra=10^4$ simulation. Most of the plots are similar to the 2D case, but some of the derivatives have been dominated by outliers and have unrecognizable features.

**Figure 15:**
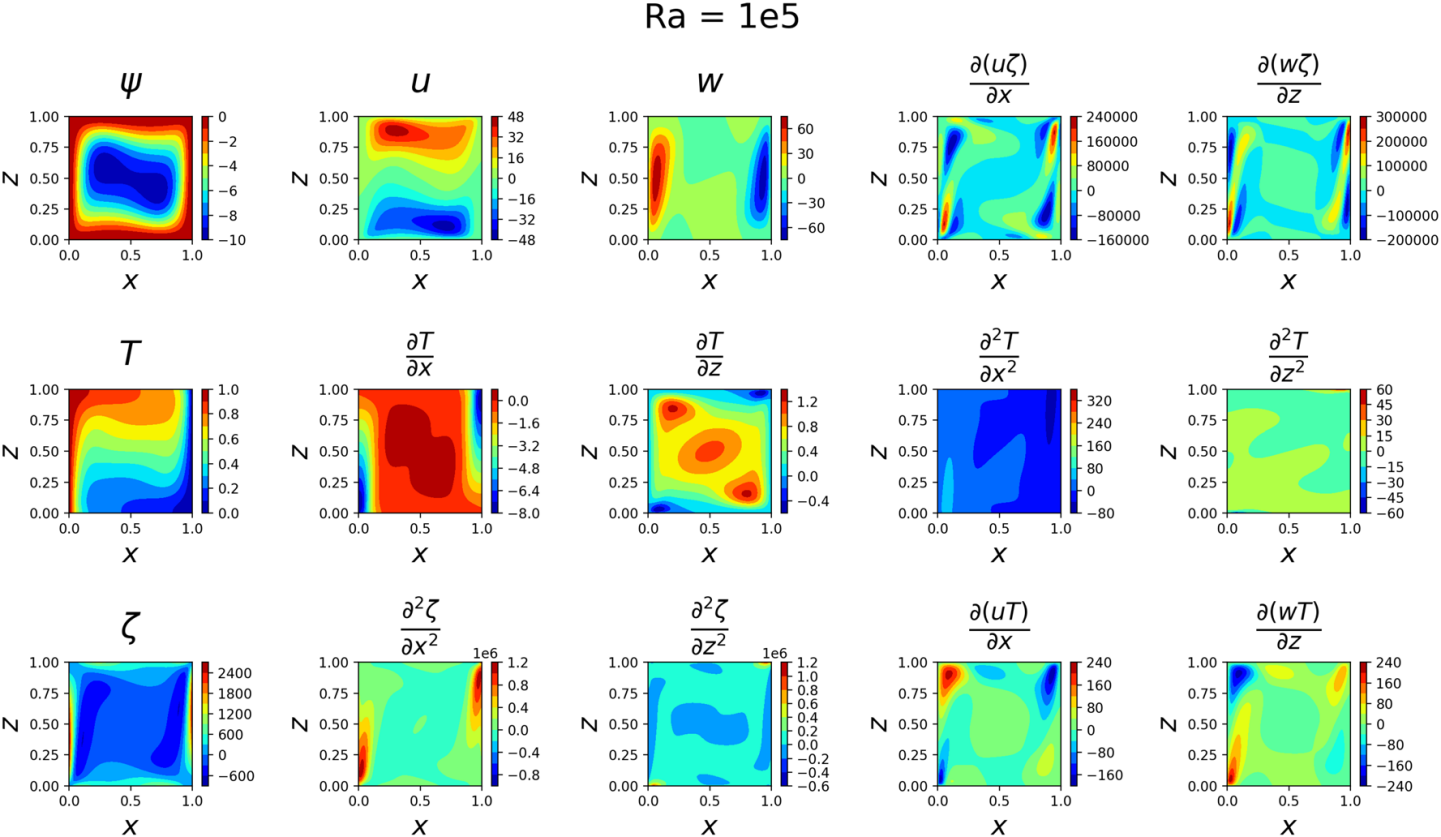
Outputs of 3D $Ra=10^5$ simulation. They continue the trend of matching the 2D results except for the derivatives that show no obvious features.

**Figure 16:**
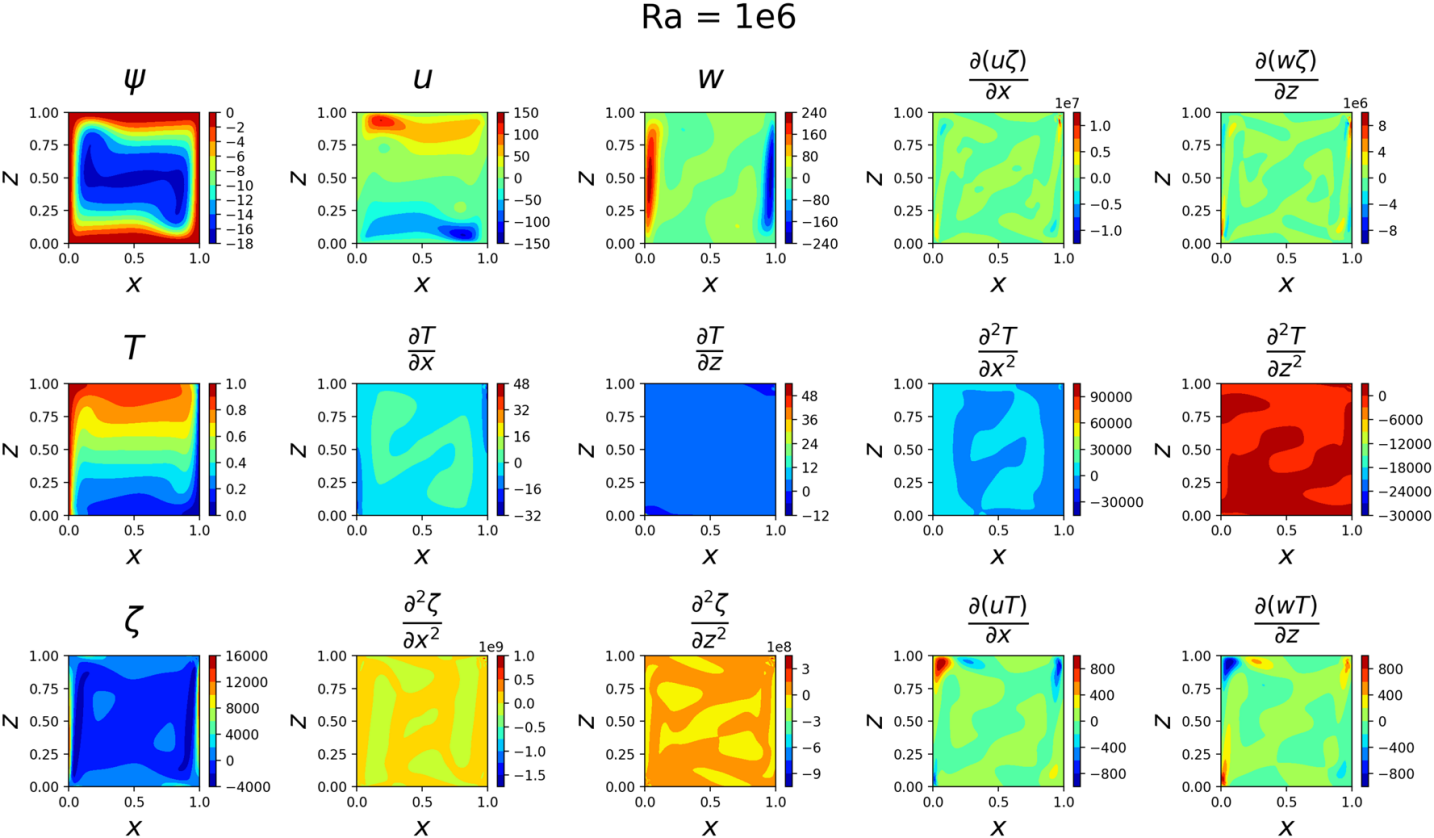
Outputs of 3D $Ra=10^6$ simulation. The few plots with distinguishable information match the 2D case, but most of the outputs contain tiny regions with large values near the walls that cause the rest of the plot to have no visible features. These tiny regions seem to underscore shortcomings that the PINN did not properly learn.

**Figure 17:**
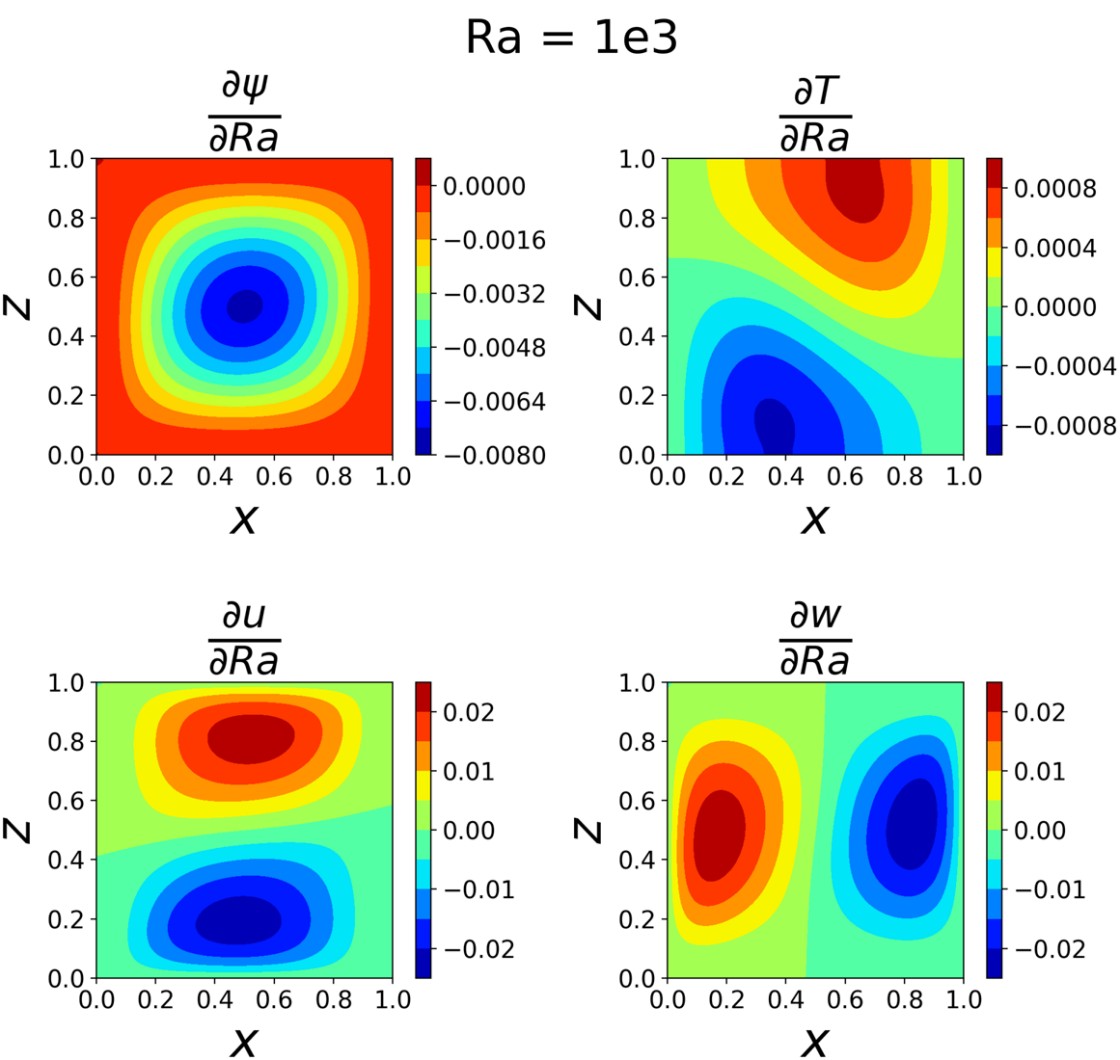
Partial derivatives of $\psi$, *T*, *u*, and *w* with respect to *Ra* when $Ra=10^3$. The features are visually similar to those from the standard $Ra=10^{3}$ outputs.

**Figure 18:**
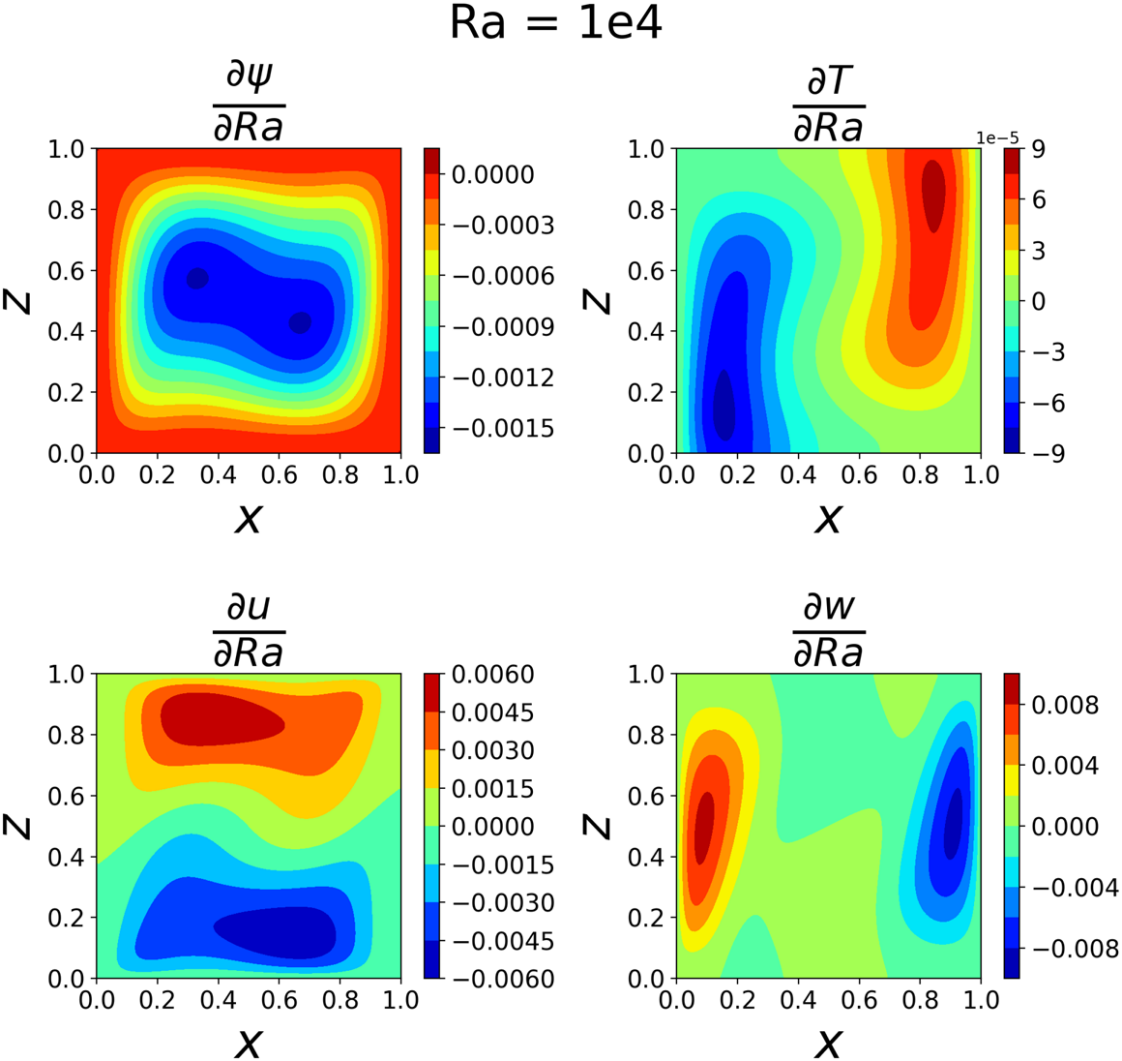
Partial derivatives of $\psi$, *T*, *u*, and *w* with respect to *Ra* when $Ra=10^4$. $\psi$ can be seen splitting into two regions and the temperature derivative shows more elongated regions than in $Ra=10^{3}$.

**Figure 19:**
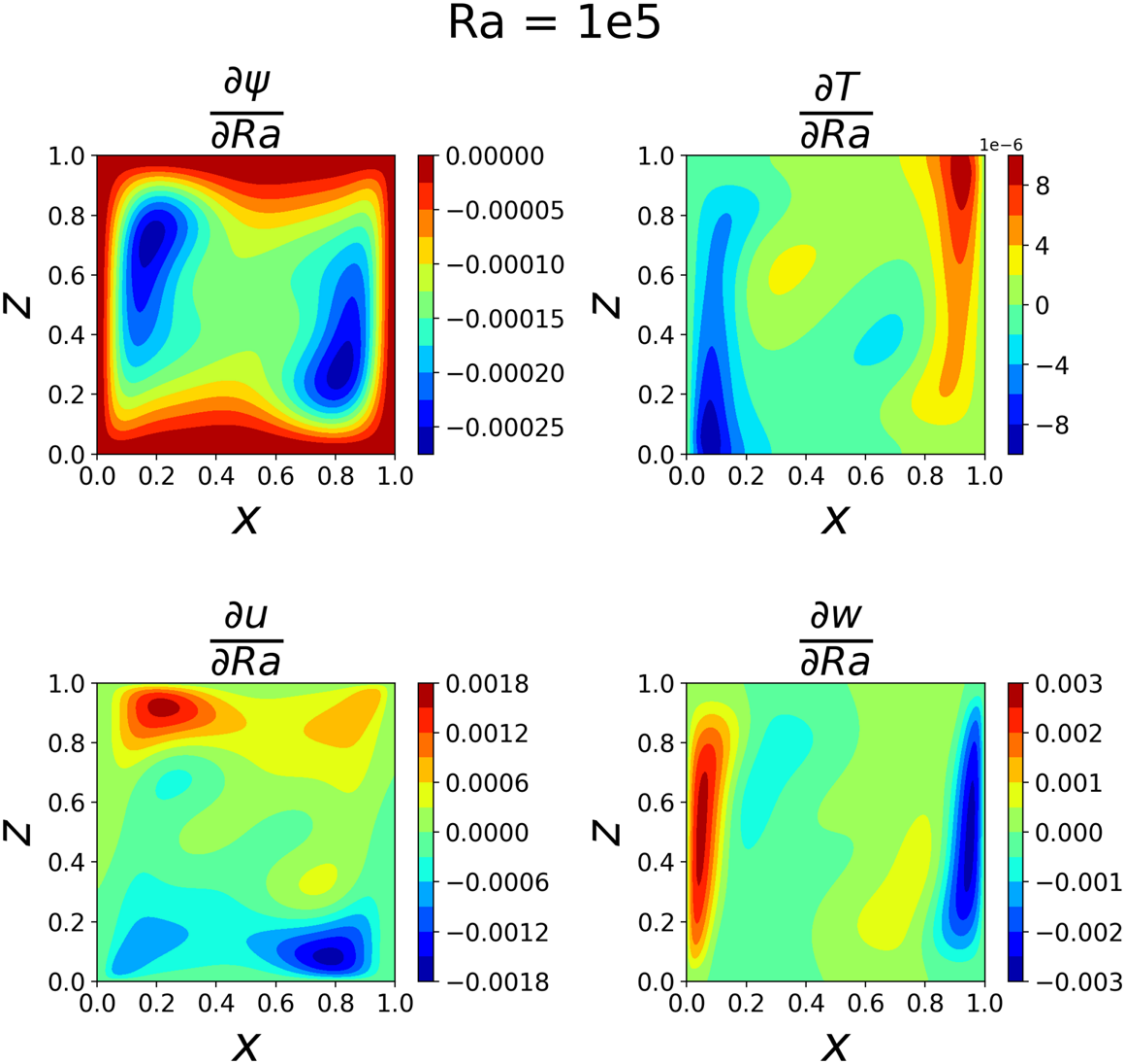
Partial derivatives of $\psi$, *T*, *u*, and *w* with respect to *Ra* when $Ra=10^5$. The *u* derivative now has two regions of increasing magnitude and two regions of decreasing magnitude.

**Figure 20:**
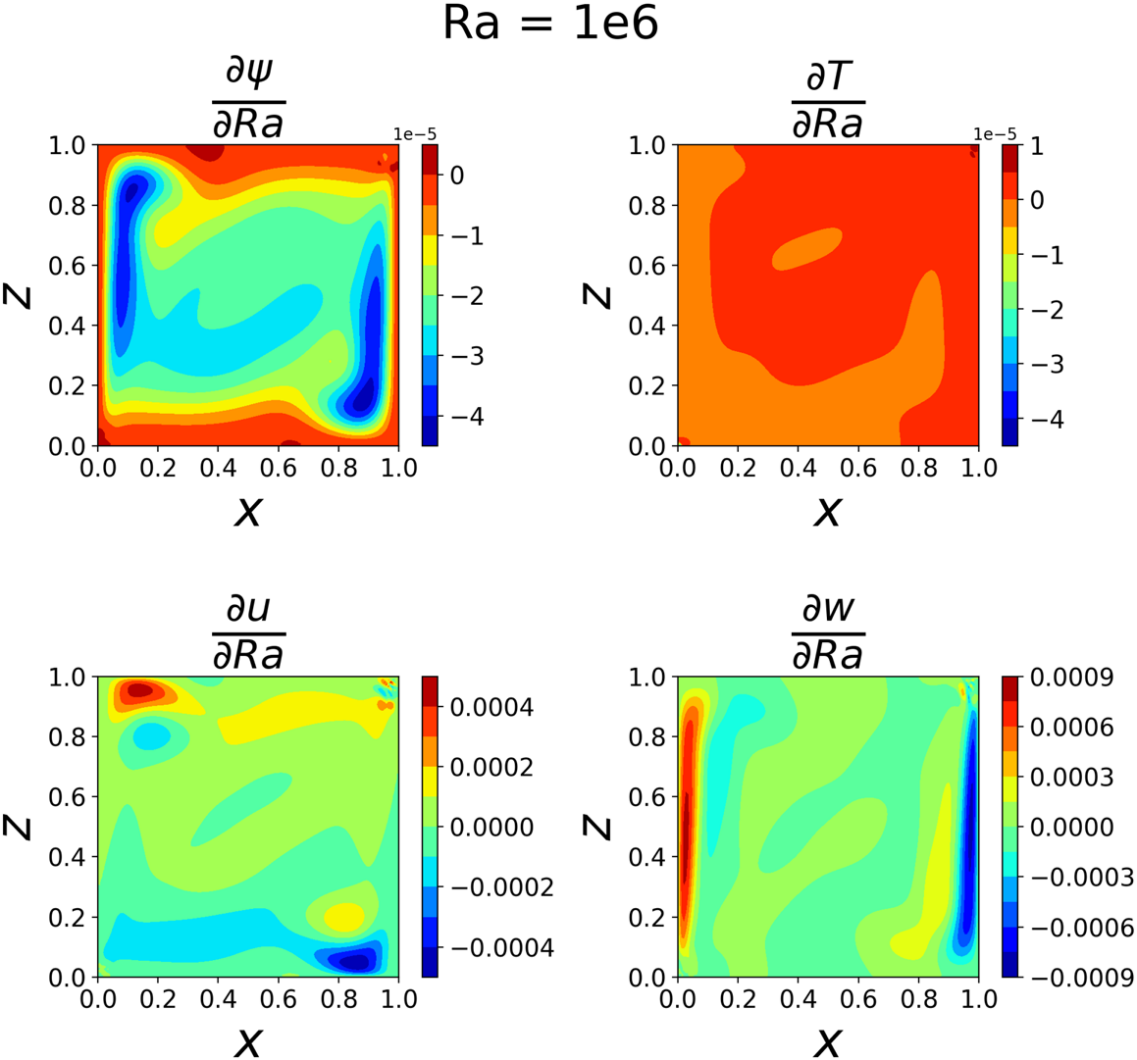
Partial derivatives of $\psi$, *T*, *u*, and *w* with respect to *Ra* when $Ra=10^6$. Similarly to Fig. 16, tiny pockets of high magnitudes show up in the corners that indicate the PINN was not able to successfully learn the solution at $Ra=10^{6}$.

**Figure 21:**
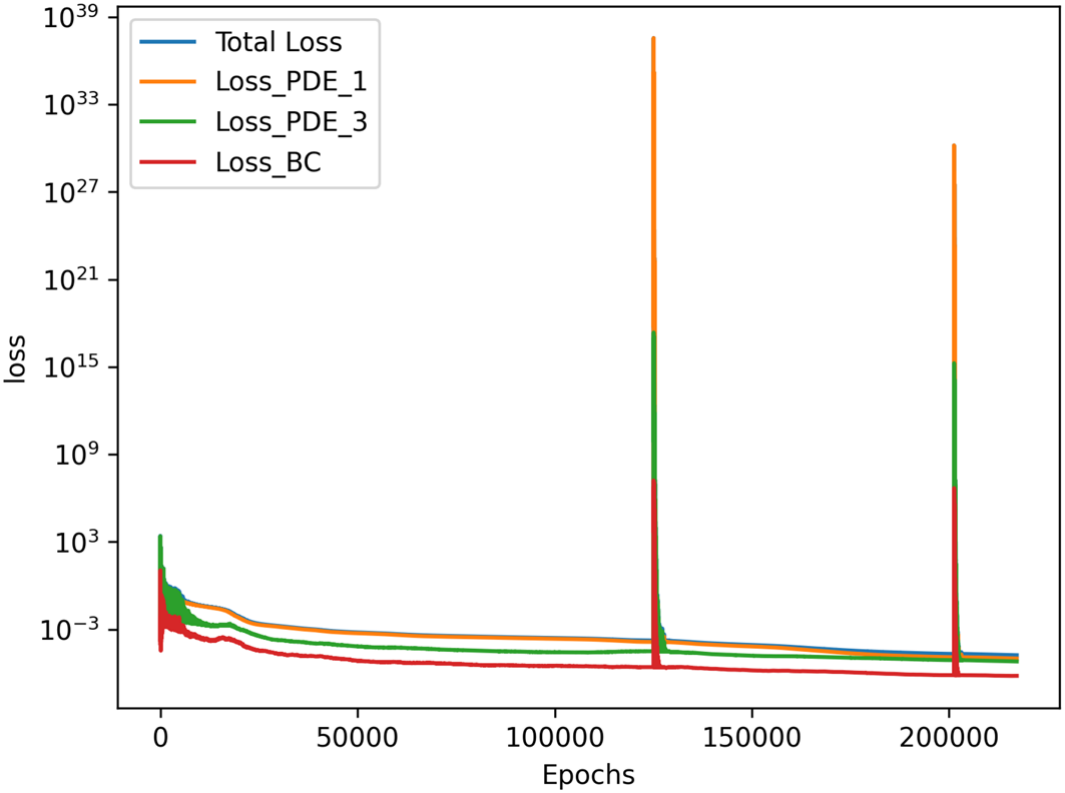
Loss graph of 3D simulation across *Ra* values from $10^{3}$ to $10^{6}$.

The results of the 3D simulation over (*x*, *z*, *Ra*) were shown at the same four *Ra* values as the 2D cases. The results are tabulated in Table 3, and their plots are shown in Figs. 13, 14, 15and 16. The 3D simulation ran for 3 days, 1 hour, and 58 minutes to reach a loss on the order of $10^{-6}$ with predictions within $1\%$ for each of the reference values^11^ except *u* at $Ra=10^{6}$. The gradients of $\psi$, *T*, *u*, and *w* with respect to *Ra* at $Ra = 10^{3}$, $10^{4}$, $10^{5}$, and $10^{6}$ are shown in Figs. 17, 18, 19 and 20. The 3D model training was paused twice to inspect the training progress due to the increased training time. This caused the BFGS optimizer to be reinitialized and resulted in the two brief spikes in training loss shown in the loss plot in Fig. 21. However, these did not affect the model’s ability to converge.

**Table 3:** Comparison between Ref.^11^ and our PINN predicted values for the 3D Cavity.

	$Ra=10^3$	$Ra=10^4$	$Ra=10^5$	$Ra=10^6$
$|\psi |_{ref}$ location (*x*, *z*)	(0.5, 0.5)	(0.5, 0.5)	(0.5, 0.5)	(0.5, 0.5)
$|\psi |_{ref}$	1.174	5.071	9.111	16.32
$|\psi |_{pred}$	1.169	5.068	9.140	16.16
$u_{ref}$ location (*x*, *z*)	(0.5, 0.813)	(0.5, 0.823)	(0.5, 0.855)	(0.5, 0.850)
$u_{ref}$	3.649	16.178	34.73	64.63
$u_{pred}$	3.630	16.167	34.78	62.77
$w_{ref}$ location (*x*, *z*)	(0.178, 0.5)	(0.119, 0.5)	(0.066, 0.5)	(0.0379, 0.5)
$w_{ref}$	3.697	19.617	68.59	219.36
$w_{pred}$	3.681	19.606	68.68	218.53

In Table 3, the prediction for *u* at $Ra=10^{6}$ stands out as most inaccurate in comparison to the other predictions when compared to the reference values. In addition to having this weak prediction, the 3D PINN was overall less accurate to the reference values than the prediction accuracy of the four 2D models.

The partial derivative plots Figs. 17, 18, 19 and 20 offer unique perspectives to the solution. These derivatives show how the solution changes as a function of *Ra*, but they could also be taken with respect to any variable. This could be useful in a design scenario, such as if an engineer wanted to find the *Ra* where *u* only had one local maximum in order to place a sensor when running experiments.

The regions of large magnitudes in the partial derivative plots match up well with the features that change the most between the standard output plots at each *Ra* value. This supports the novel goal of using a PINN to leverage predictive power at any location in the training domain while also being able to view what is causing new features to form as *Ra* varies.

**Figure 22:**
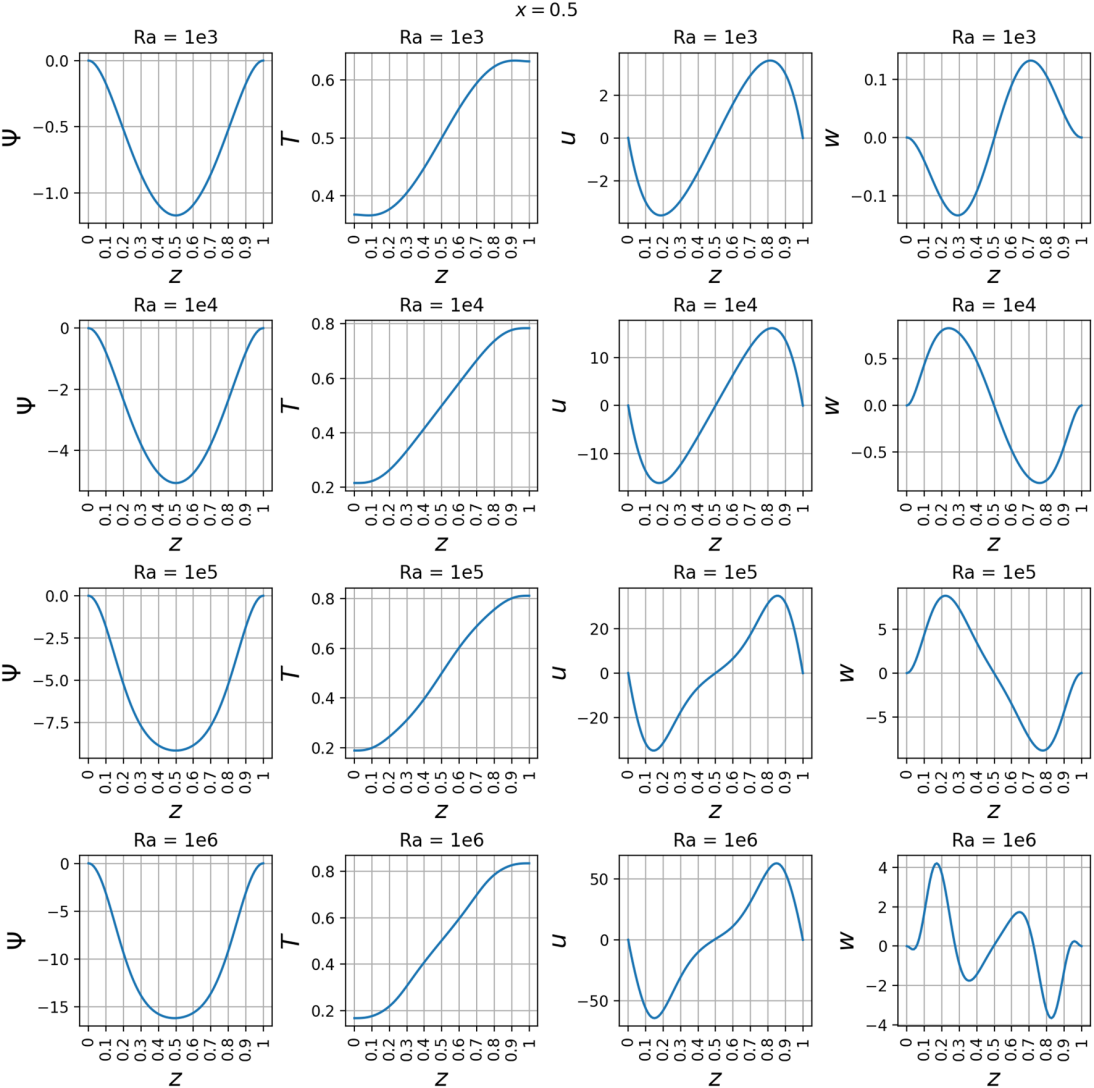
$\psi$, *T*, *u*, and *w* at the vertical mid-plane where $x=0.5$ and *z* varies from 0 to 1. These are plotted at *Ra* values of $10^3$, $10^4$, $10^5$, and $10^6$. $\psi$, *T*, and *u* keep similar shapes across *Ra* whereas *w* has significant changes from $Ra=10^3$ to $Ra=10^4$ and from $Ra=10^5$ to $Ra=10^{6}$.

**Figure 23:**
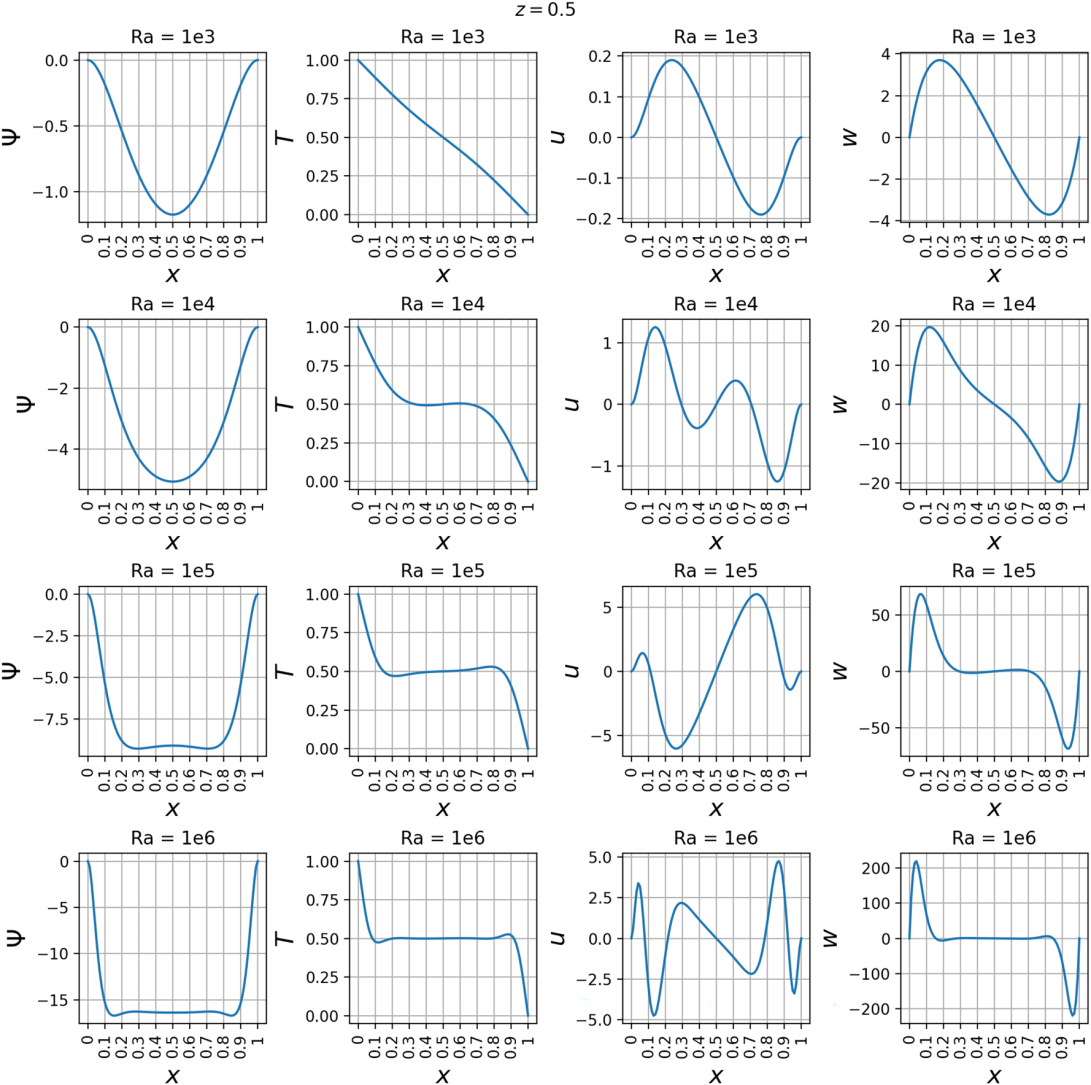
$\psi$, *T*, *u*, and *w* at the horizontal mid-plane where $z=0.5$ and *x* varies from 0 to 1. These are plotted at *Ra* values of $10^3$, $10^4$, $10^5$, and $10^6$. *T* appears almost linear at $Ra=10^{3}$.

**Figure 24:**
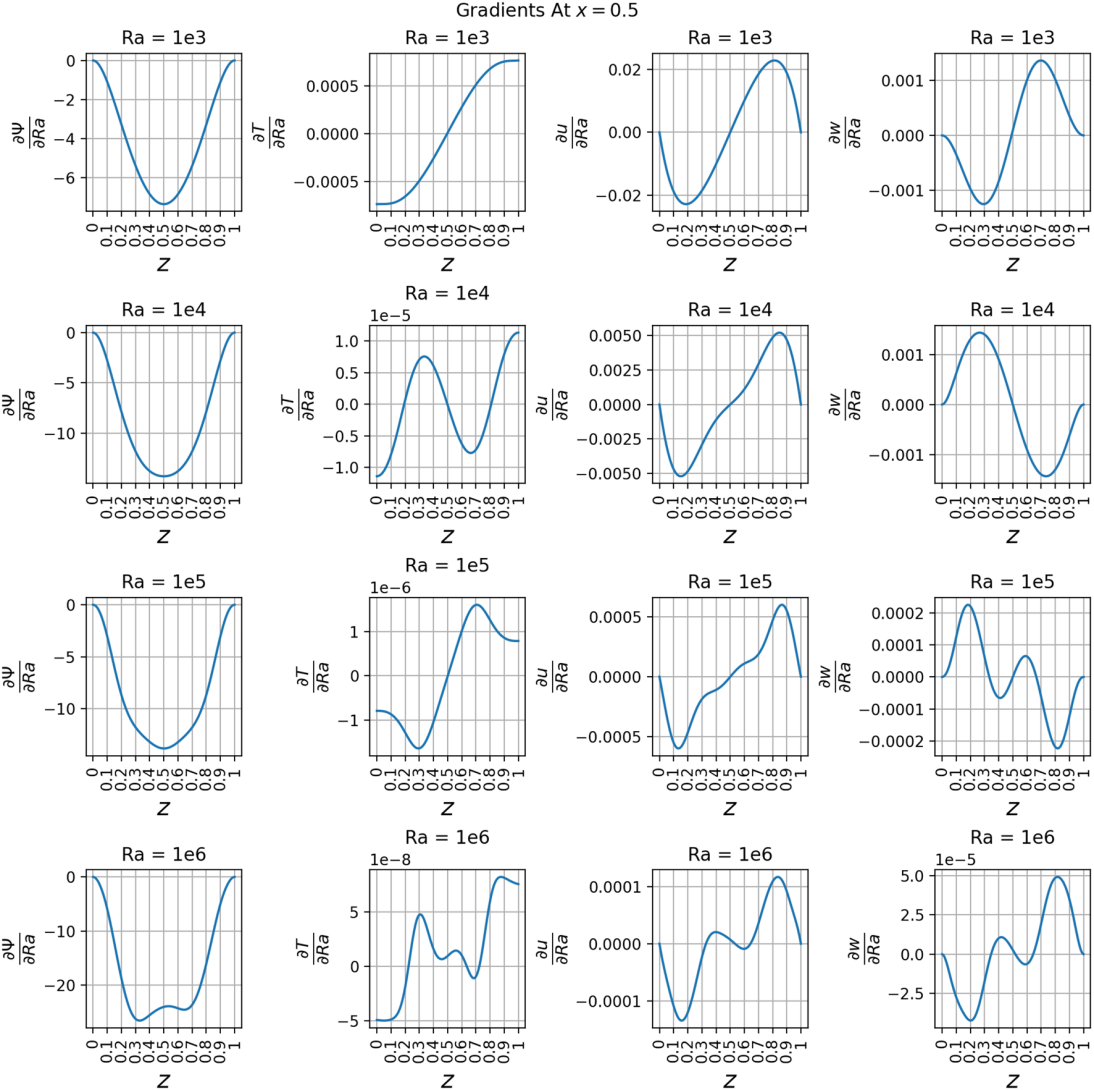
Derivatives of $\psi$, *T*, *u*, and *w* with respect to *Ra* at the vertical mid-plane where $x=0.5$ and *z* varies from 0 to 1. These are plotted at *Ra* values of $10^3$, $10^4$, $10^5$, and $10^6$.

**Figure 25:**
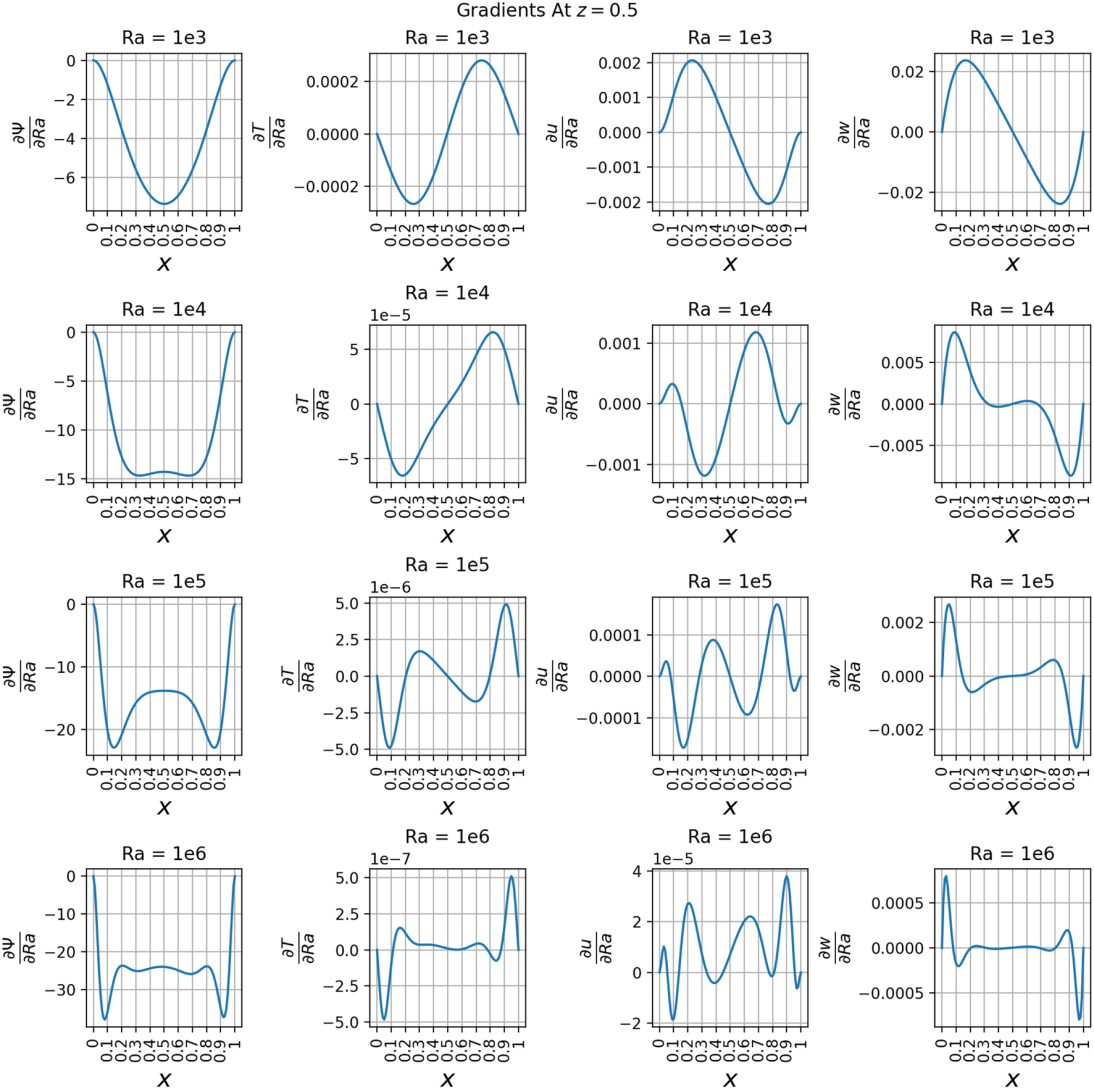
Derivatives of $\psi$, *T*, *u*, and *w* with respect to *Ra* at the horizontal mid-plane where $z=0.5$ and *x* varies from 0 to 1. These are plotted at *Ra* values of $10^3$, $10^4$, $10^5$, and $10^6$.

The line plots in Fig. 22 along *z*-axis at the vertical mid-plane show the increase in the variation of state variables $\psi$, *T*, *u*, and *w* as a function of Rayleigh number. To demonstrate the three-dimensional spatial distribution, Fig. 23 plots the variation of the same state variables along *x*-axis at the horizontal mid-plane for Rayleigh numbers (Ra = $10^3, 10^4, 10^5, 10^6$). The line plots of the derivatives of the state variables $\psi$, *T*, *u*, and *w* with respect to the Rayleigh number are shown along *z* axis at the *x* mid-plane in Fig. 24 and along the *x*-axis at the *z* mid-plane in Fig. 25. Results show the sharp change in the functional variation, which indicates a stronger coupling between the inertial (viscous) and thermal (buoyant) forces as the Rayleigh number increases from $10^3$ to $10^6$

.

## Discussion

The simulations were all able to match their expected values^11^, which were the value of the stream function $(|\psi |)$ at the center, the maximum value of *u*, and the maximum value of *w*. The 2D cases matched their reference values^11^ more closely than the 3D data did. Both the 2D and 3D results were within the error estimates from the literature^11^. In addition, the contour plots for each value, *u*, *w*, $\psi$, *T*, and $\zeta$, matched what is reported in literature^11,12^.

The comparison of *Nu* in Table 2 shows additional metrics where the PINN results matched the published data within $\pm 0.2\%$. Note that higher Ra solutions are computationally more demanding than the lower Ra cases. For example, $Ra=10^6$ required an order of magnitude more CPU time than $Ra = 10^3$ solution. Furthermore, the quantitative comparisons in Figs. 10, 11 and 12 provide further verification of the PINN’s viability showing that the PINN results match within $\pm 0.1\%$ with the reported data even for higher $Ra=10^{6}$ where the flow is increasingly convection dominated.

One feature of the results that stood out was how the necessary training time was short and similar for $Ra=10^3$ and $Ra=10^4$, but it significantly increased for $Ra=10^5$ and $Ra=10^6$. One interesting finding, found during hyperparameter tuning, was that by stopping the latter two simulations early in training, it was noticeable that the PINN was learning what seemed to be somewhat close to the correct solution, but with everything multiplied by $-1$. Eventually, the model did converge to the right solution, but it took much longer for these cases. This indicates the model was likely stuck in a deep local minimum before it converged to the correct solution.

The 3D case took much longer to train than any of the 2D cases. Upon inspecting intermediary training steps, the issue that the 2D $Ra=10^5$ and $Ra=10^6$ cases showed of initially converging to an incorrect local minimum was not present in the 3D simulation. This likely indicates that learning using values close to $Ra=10^3$ and $Ra=10^4$, where the solution converges much more reliably, helped to remove some of the difficulty learning for the higher values of *Ra*. However, despite training for significantly longer than any of the 2D cases, the 3D model showed overall slightly weaker results than the 2D cases at each *Ra* test case. These performance decreases when using the 3D case indicate that individuals should be cautious when selecting to use high-dimensionality PINN models for forward simulations without known data.

Despite the relative success of the 3D simulation, some of the figures show where the network faced some limitations. This is easily noticed for the $Ra=10^6$ case in Fig. 16, where any result showing vorticity contains a point in the top-right corner of significantly increased magnitude, which dominates the rest of the features of the plot. In comparison, the 2D solution in Fig. 5 shows results that contain much more noticeable patterns and features while not having that strong peak in the corner. Similarly, Fig. 16 contains a hook in the top-right corner of the ${{\partial }(uT)}\over {{\partial }z}$ plot that is not present in the 2D solution in Fig. 5. The 2D solution instead contains a smooth contour in that location. However, the magnitude of the contour is similar to the magnitude of the contour in Fig. 16. This sharp gradient in the corners was also prevalent in Fig. 20, which strongly indicates that more training points may be necessary to constrain the solution in these regions. Other potential solutions at these high *Ra* values could include using a more rich network and more training epochs.

The primary goal of the paper was to confirm the validity of the PINN code for CFD, and the models were able to successfully meet these expectations. The 2D codes performed well at each *Ra*, and the 3D codes were also able to produce adequate results at each *Ra*. The runtimes for $Ra=10^{3}$ and $Ra=10^4$ were under an hour, and the runtimes for $Ra=10^{5}$ and $Ra=10^6$ were on the order of hours. The runtime of 3 days for the 3D model was much larger than the 4 hours for the 2D models with the same number of epochs. We suspect that this could be an inefficiency related to using very close to the maximum amount of memory, dividing by the vector of *Ra* values instead of a single constant, perhaps the increased number of training points was not perfectly evaluated in parallel, or maybe the 3rd input impacted backpropagation. In comparison, the technology of 15 years ago could achieve results in around 10 minutes for $Ra=10^3$ and 3 hours for $Ra=10^6$^12^. Even using modern hardware, our 2D PINN simulations were unable to offer any improvement in computational time. For direct forward 2D simulations without known data, PINNs did not show an improvement over conventional CFD methods.

The secondary goal of this paper was to explore the use of a 3D parameter space. This model was able to produce reasonable solutions for any $10^{3}\le Ra \le 10^{6}$ in a much longer time than it took to train the 2D $Ra=10^6$ case. This training time showed that a model with a larger parameter space could incur extensive resource costs. However, the 3D case was able to provide derivatives of the outputs with respect to *Ra*, which clearly showed how the features of the solution were changing as the value of *Ra* increased. These derivatives could be quickly evaluated in seconds for testing various *Ra* values.

This unique capability of PINNs over conventional CFD programs could prove very useful for identifying the development of certain flow characteristics as the input parameters change. The abilities of PINNs to learn higher-dimensionality parameter spaces and their performance increase when learning from data^19^ indicate that they could be paired with traditional CFD methods very effectively. The CFD could provide high-accuracy training points, and the PINNs could be trained to interpolate solutions between those points from inputs in a higher-dimensionality parameter space.

## Conclusion

Overall, the PINN was able to successfully run the forward simulation of the 2D cavity problem for all tested values of *Ra*. The solutions are visibly similar contour profiles and for select plots matched quantitatively within $\pm 0.2 \%$ with previously reported data. Furthermore, the 3D simulation was also able to successfully match the known results at the four tested values of *Ra* while only needing to train one model on the increased parameter space. However, 3D model showed some shortcomings in the $Ra = 10^6$ case indicatingroom for improvement for increased nonlinearly coupled thermofluid problems. The 3D model also showcased the insights offered by learning a higher-dimensionality parameter space through its unique ability to use this parameter space to create derivatives that would normally be inaccessible without running multiple independent simulations. Importantly, all cases were run without leveraging known data which was later used only to validate the PINN predicted results.

With the code for the 2D and 3D cases validated and the unique opportunities presented by the 3D case, future work should continue to focus on these unique opportunities. Further validation work remains to be done for confirming how PINNs compare the traditional CFD methods in computational speed and problem complexity (${e.g.}$, turbulence), but PINNs certainly have a place working alongside traditional methods to learn from CFD results and expand upon them to provide design guidance with unique solutions and perspectives to practical problems.

## Data Availability

The Python codes with models that generated datasets presented in this paper are available in the GitHub repository [https://github.com/cmcdevitt2/ThermalCavity].
